# Differences in coreceptor specificity contribute to alternative tropism of HIV-1 subtype C for CD4^+^ T-cell subsets, including stem cell memory T-cells

**DOI:** 10.1186/s12977-014-0097-5

**Published:** 2014-11-12

**Authors:** Kieran Cashin, Geza Paukovics, Martin R Jakobsen, Lars Østergaard, Melissa J Churchill, Paul R Gorry, Jacqueline K Flynn

**Affiliations:** Center for Biomedical Research, Burnet Institute, Melbourne, 3004 Australia; Department of Microbiology and Immunology, University of Melbourne, Melbourne, 3010 Australia; Burnet Institute Flow Cytometry Core Facility, Melbourne, 3004 Australia; Department of Biomedicine, Aarhus University, Aarhus, 237551 Denmark; Department of Infectious Diseases, Aarhus University, Aarhus, 237551 Denmark; Department of Medicine, Monash University, Melbourne, 3004 Australia; Department of Microbiology, Monash University, Melbourne, 3010 Australia; Department of Infectious Diseases, Monash University, Melbourne, 3004 Australia

**Keywords:** HIV-1, Subtype C, T-cell, CD4^+^, T_SCM_

## Abstract

**Background:**

CD4^+^ memory T-cells are a major target for infection by HIV-1, whereby latent provirus can establish and endure suppressive antiretroviral therapies. Although HIV-1 subtype C strains (C-HIV) account for the majority of HIV-1 infections worldwide, the susceptibility of CD4^+^ memory T-cells to infection by CCR5- (R5) and CXCR4-using (X4) C-HIV is unknown. Here, we quantified the susceptibility of naïve and memory CD4^+^ T-cell subsets, including stem cell memory T-cells (T_SCM_), to infection by HIV-1 subtype C (C-HIV) strains from treatment-naïve subjects who progressed from chronic to advanced stages of disease whilst either maintaining CCR5-using (R5) viruses (subjects 1503 and 1854), or who experienced emergence of dominant CXCR4-using (X4) strains (subject 1109).

**Findings:**

We show that R5 and X4 C-HIV viruses preferentially target memory and naïve CD4^+^ T-cell subsets, respectively. While T_SCM_ were susceptible to infection by both R5 and X4 C-HIV viruses, the proportion of infected CD4^+^ T-cells that were T_SCM_ was higher for R5 strains. Mutagenesis studies of subject 1109 viruses established the V3 region of *env* as the determinant underlying the preferential targeting of naïve CD4^+^ T-cells by emergent X4 C-HIV variants in this subject. In contrast, the tropism of R5 C-HIV viruses for CD4^+^ T-cell subsets was maintained from chronic to advanced stages of disease in subjects 1503 and 1854.

**Conclusions:**

This study provides new insights into the natural history of tropism alterations for CD4^+^ T-cell subsets by C-HIV strains during progression from chronic to advanced stages of infection. Although not preferentially targeted, our data suggest that T_SCM_ and other memory CD4^+^ T-cells are likely to be viral reservoirs in subjects with X4 C-HIV infection.

**Electronic supplementary material:**

The online version of this article (doi:10.1186/s12977-014-0097-5) contains supplementary material, which is available to authorized users.

## Findings

CD4^+^ T-cells are targets for infection by human immunodeficiency virus type 1 (HIV-1), whereby latent, replication-competent provirus can persist despite suppressive antiretroviral therapies [1]. Following exposure to antigen, naïve CD4^+^ T-cells (T_N_) proliferate and differentiate into antigen-specific effector cells or self-renewing memory T-cells [2,3]. Memory CD4^+^ T-cell subsets include long-lived central memory T-cells (T_CM_), transitional memory (T_TM_) and shorter-lived effector memory T-cells (T_EM_) [4,5]. Recently, a new memory T-cell subset was described in CD4^+^ and CD8^+^ T-cell populations in humans [6-8]. These cells, termed stem cell memory T cells (T_SCM_), account for approximately 2–4% of the CD4^+^ T-cell population in HIV-seronegative individuals, and are the longest-lived memory CD4^+^ T-cell subset [8,9]. Importantly, T_SCM_ contribute to the persistent cellular HIV-1 reservoir [6].

HIV-1 entry into cells is mediated by the interaction between the viral envelope protein complex (Env) and cell-surface CD4, and then with a chemokine coreceptor, either CCR5 or CXCR4 (reviewed in [10]). The ability of HIV-1 to engage CCR5, CXCR4 or either coreceptor for entry is a major determinant of cellular tropism, and is used to classify viruses as R5, X4 or R5X4, respectively [11].

Although HIV-1 subtype C (C-HIV) strains account for >50% of HIV-1 infections worldwide and exhibit pathogenicity and tropism alterations that are distinct from other HIV-1 subtypes [12], at present there are few studies describing the susceptibility of CD4^+^ T-cell subsets to infection by clinical R5 C-HIV strains, and none concerning clinical X4 C-HIV isolates. Given recent reports that have described increased incidence of X4 C-HIV strains across multiple cohorts, particularly from patients with late-stage disease (up to 52%) [12-17], this lack of knowledge has implications for HIV/AIDS cure strategies, including the widely discussed use of histone deacetylase inhibitors to activate transcription of latent provirus and purge CD4^+^ memory T-cell reservoirs.

Since changes in HIV-1 cellular tropism may influence the size and composition of viral reservoirs, here we sought to quantify and compare the susceptibility of naïve and memory CD4^+^ T-cell subsets, including T_SCM_, to infection by clinical X4 and R5 C-HIV strains from patients who either maintained R5 viruses or experienced emergence of X4 strains during progression from chronic to advanced stages of C-HIV infection.

### Experimental procedures

We utilized X4 (n = 2) and R5 (n = 15) Envs cloned from longitudinal plasma samples of three treatment-naïve C-HIV infected subjects (1503, 1854 and 1109) from the recently described MUSH cohort [12] who, over a 3-year sampling period, showed clinical and immunological evidence of progression from chronic to advanced stages of infection [12,17] (Table 1). Subjects 1503 and 1854 exclusively harboured R5 viruses in plasma samples provided at enrolment (E), 1-year later (I; intermediate) and 3-years after enrolment (F; final), whereas subject 1109 experienced a switch from dominant R5 viruses in enrolment and intermediate plasma samples to dominant X4 strains in the final plasma sample [12] (Table 1).

**Table 1:** **Subject 1503, 1854 and 1109 Envs**

**Subject**	**Plasma sample**	**CD4** ^**+**^ **T-cell count (cells/μL)**	**Viral Load (RNA copies/ml)**	**Env ID**	**Coreceptor usage**
**1503**	E	396	5.73	11	R5
				13	R5
	I	168	5.69	7	R5
				11	R5
	F	259	5.71	7	R5
				8	R5
**1854**	E	435	5.61	8	R5
				10	R5
	I	279	6.57	4	R5
				20	R5
	F	272	5.67	4	R5
				8	R5
**1109**	E	367	5.54	10	R5
				42	R5
	I	402	5.59	3	R5
	F	169	5.67	30	X4
				39	X4
	-	-	-	F-30-E10V3	R5

The CD4^+^ T-cell counts and plasma viral loads for these subjects have been reported previously, and are summarized again here to assist in the interpretation of the CD4^+^ T-cell subset tropism data [12]. Plasma viral load values are shown as log_10_. Envs were cloned directly from plasma samples provided at enrolment (E), 1-year later (I; intermediate) and 3-years after enrolment (F; final). Env coreceptor usage was determined by infecting NP2 or U87 cells expressing CD4 and either CCR5 or CXCR4 with Env-pseudotyped luciferase reporter viruses [12]. The 1109-F-30-E10V3 Env was generated by mutating 1109-F-30 to express the V3 region of 1109-E-10 [12,17]. Env ID, HIV-1 Env identification number as described in [12].

To determine the CD4^+^ T-cell subset tropism for each of these Envs, we utilized an *in vitro* multi-parameter flow cytometry-based infection assay, described in our recent studies [18,19] and in Additional file 1. Briefly, peripheral blood CD4^+^ T-cells isolated from four HIV-seronegative donors were infected with single-round Env-pseudotyped GFP reporter viruses, then HIV-1 infection was determined by GFP positivity and the distribution of infection among CD4^+^ T-cell subsets was determined using antibodies specific for cell-surface markers; T_N_ (CD45RO^−^CCR7^+^CD27^+^), T_SCM_ (CD45RO^−^CCR7^+^CD27^+^CD95^+^CD122^+^), T_CM_ (CD45RO^+^CCR7^+^CD27^+^), T_TM_ (CD45RO^+^CCR7^−^CD27^+^), T_EM_ (CD45RO^+^CCR7^−^CD27^−^) and T_EMRA_ (CD45RO^−^CCR7^−^CD27^−^) The fluorochrome labeled flow cytometry antibodies used in this study are described in Additional file 1: Table S1. Our T-cell subset gating strategy, which we have described previously [18], is illustrated in Additional file 1: Figure S1.

GFP reporter viruses pseudotyped with well-characterized T-cell tropic B-HIV Envs JR-CSF (R5), HXB2 (X4) and Macs1-Spleen12 (R5X4) were used as controls [19-22]. CD4^+^ T-cells were infected with 3000 infectious units of B- and C-HIV R5 viruses, and 1250 infectious units of B- and C-HIV R5X4 and X4 viruses, which we determined here (data not shown) and previously [18,19] to be virus inoculums that ensure infections within the linear range.

### R5 and X4 C-HIV Envs exhibit alternative CD4^+^ T-cell subset tropism profiles

**Figure 1:**
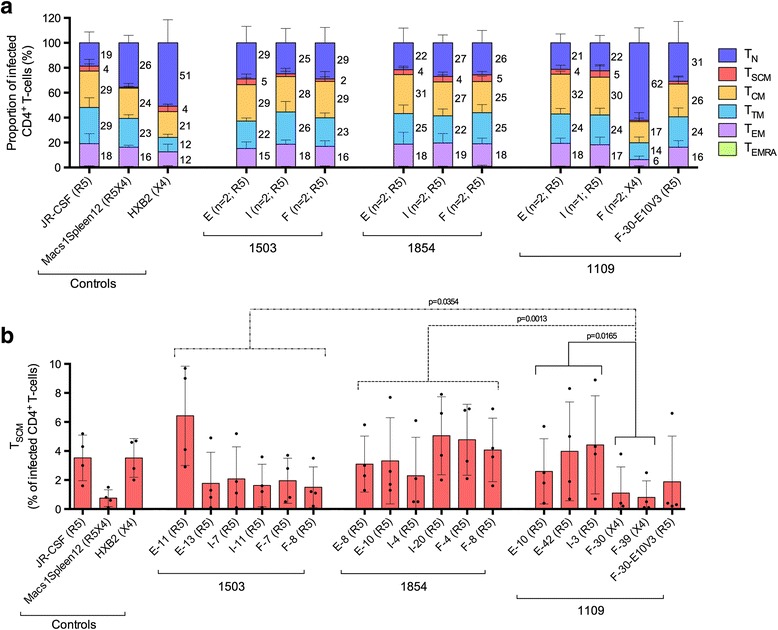
**Proportion of CD4** ^**+**^ **T-cell subsets infected by Env-pseudotyped GFP reporter viruses. (a)** Values represent the percentage of infected CD4^+^ T-cells that belong to the indicated subset; naïve (T_N_, dark blue), stem cell memory (T_SCM_, red), central memory (T_CM_, yellow), transitional memory (T_TM_, light blue), effector memory (T_EM_, purple) and effector memory RA (T_EMRA_, green). Values were averaged across four HIV-seronegative donors. Error bars represent the standard deviation. CD4^+^ T-cells were infected with GFP reporter viruses pseudotyped with (i) subtype C HIV-1 Envs isolated from plasma samples of subjects 1503, 1854 and 1109 provided at enrolment (E), 1-year later (I; intermediate) and 3-years after enrolment (F; final), or (ii) subtype B HIV-1 control Envs. Text in parentheses indicates the number of Envs isolated per plasma sample and/or Env coreceptor usage; CCR5-specific (R5), CXCR4-specific (X4), uses CCR5 or CXCR4 (R5X4). **(b)** Values represent the percentage of infected CD4^+^ T-cells that are T_SCM_ for each Env-pseudotyped GFP reporter virus per donor (individual dots). Columns display the mean and standard deviation. Statistical analyses were performed using Mann Whitney tests, p-values ≤0.05 were considered significant.

Prior to infection we first showed that the proportion of each CD4^+^ T-cell subset (Additional file 1: Figure S2) and the proportion of cells expressing CCR5 and CXCR4 (Additional file 1: Table S2) was similar between donors, and was consistent with the findings of previous studies [10,18,19,23,24]. R5 C-HIV Envs mediated levels of CD4^+^ T-cell infection (mean 1% ± standard deviation 0.4% of infected CD4^+^ T-cells) similar to JR-CSF (1 ± 0.1%), and preferentially infected memory T-cells; T_N_ (21.5 ± 8.9%), T_SCM_ (3.3 ± 2.6%), T_CM_ (24.7 ± 5.3%), T_TM_ (20.1 ± 4.7%), T_EM_ (14.9 ± 4.8%) and T_EMRA_ (0.6 ± 0.4%) (Figure 1a). X4 C-HIV Envs mediated levels of CD4^+^ T-cell infection (3.5 ± 1.8%) similar to HXB2 (1.7 ± 0.9%), and preferentially infected naïve T-cells; T_N_ (62.1 ± 12.1%), T_SCM_ (1.1 ± 1.7%), T_CM_ (16.9 ± 7.3%), T_TM_ (13.5 ± 4.6%), T_EM_ (5.7 ± 2.8%) and T_EMRA_ (0.6 ± 0.7%), (Figure 1a). The preferential infection of memory and naive T-cells by R5 and X4 C-HIV Envs, respectively, could be influenced by the variation in the proportion of cells that express CCR5 and CXCR4 (Additional file 1: Table S2) as CD45RO^−^ cells had a higher proportion of CD4^+^ T-cells expressing CXCR4 compared to CD45RO^+^ cells, which had higher proportions of CD4^+^ T-cells expressing CCR5. Together, these data suggest that, within these subjects, C-HIV tropism for CD4^+^ memory and naïve T-cells is constrained by Env coreceptor specificity.

### Env determinants underlying the linkage between C-HIV coreceptor specificity and CD4^+^ T-cell tropism alterations

To better understand the underlying Env determinants of C-HIV tropism for CD4^+^ T-cell subsets, we quantified the susceptibility of CD4^+^ T-cells isolated from peripheral blood of four HIV-seronegative donors to infection by GFP reporter viruses pseudotyped with X4 1109-F-30 Env that was mutated to express the V3 region of the R5 1109-E-10 Env (referred to as 1109-F-30-E10V3) (Additional file 1: Table S3). Previously we utilized this Env to define the critical C-HIV V3 alterations necessary to confer CXCR4 usage [12,18]. In these studies, we confirmed that either an Ile314-Gly315 insertion or an Arg318Pro alteration in the V3 loop crown (GPGQ) motif was sufficient to confer an R5X4 phenotype, and that both of these alterations together conferred a strictly X4 phenotype. Here, the R5 1109-F-30-E10V3 Env displayed a CD4^+^ T-cell subset tropism profile that was similar to enrolment and intermediate 1109 R5 Envs, preferentially infecting memory CD4^+^ T-cells; T_N_ (30.9 ± 17.2%), T_SCM_ (2.1 ± 3.6%), T_CM_ (26.4 ± 6.5%), T_TM_ (24.4 ± 7.1%), T_EM_ (15.9 ± 5.2%) and T_EMRA_ (0.4 ± 0.4%) (Figure 1a). These findings establish (i) the V3 loop as a key determinant of CD4^+^ T-cell subset tropism for viruses from subject 1109, and (ii) the linkage between C-HIV coreceptor specificity and CD4^+^ T-cell tropism alterations.

### V3 determinants of 1109-F-30 tropism for CD4^+^ T-cell subsets are shared by the majority of CXCR4-using C-HIV Envs

Our analyses of X4 C-HIV Envs were limited to just subject 1109 because this was the only individual within our longitudinal cohort to experience the emergence of CXCR4-using strains [12]. To gauge the likelihood that our findings would translate to other CXCR4-using C-HIV strains, we analyzed every previously published R5 (n = 1653) and CXCR4-using (n = 203) C-HIV Env sequence for expression of the defined 1109-F-30 V3 determinants of CXCR4-usage and tropism for CD4^+^ T-cell subsets, namely the two amino acid insertion at positions 314–315 and/or the GPGQ crown alteration. Additional file 1: Table S4 shows that of the CXCR4-using V3 sequences, 27.1% contained a 314–315 insertion, of which 81.9% contained Ile314-Gly315 (36.5% overall), 62.6% exhibited a GPGQ alteration, of which 55.1% exhibited GRGQ (58.1% overall), and 23.2% contained both a 314–315 insertion and a GPGQ alteration, of which 84.9% exhibited both Ile314-Gly315 and GRGQ (32.4% overall). These proportions were even higher when assessing just strictly X4 C-HIV Envs, where 44.6% contained a 314–315 insertion, of which 81.8% contained Ile314-Gly315 (36.5% overall), 83.8% exhibited a GPGQ alteration, of which 69.3% exhibited GRGQ (58.1% overall), and 39.2% contained both a 314–315 insertion and a GPGQ alteration, of which 82.7% exhibited both Ile314-Gly315 and GRGQ (32.4% overall). Conversely, none of the R5 V3 sequences contained a 314–315 insertion and only 1.9% exhibited a GPGQ alteration (none of which were GRGQ). Although further functional studies are required to establish the CD4+ T-cell subset tropism properties of diverse CXCR4-using C-HIV strains, these sequence analyses suggest that a substantial proportion of previously reported X4 C-HIV Envs will likely exhibit a similar profile to that of the X4 Envs from subject 1109.

### T_SCM_ are susceptible to infection by CCR5- and CXCR4-using clinical C-HIV viruses

Recently, a new subset of CD4^+^ T-cells, T_SCM_, was found to contribute significantly to the viral reservoir during HIV-1 disease progression [6]. Here, we found that the proportion of CD4^+^ T-cells infected by X4 C-HIV Envs from subject 1109 that were T_SCM_ (1 ± 1.4%) was significantly less (by Mann Whitney test) than that of R5 C-HIV strains from subject 1109 (3.7 ± 2.9%, p = 0.0165), 1503 (2.6 ± 2.6%, p = 0.0354) and 1854 (3.8 ± 2.4%, p = 0.0013) (Figure 1b). Unexpectedly, the proportion of T_SCM_ infected by the R5 1109-F-30-E10V3 Env (1.9 ± 3.2%) was statistically similar to both R5 and X4 C-HIV isolates from subject 1109, suggesting that additional mutations in the 1109-F-30 Env are required to confer T_SCM_ entry levels alike subject 1109 R5 Envs.

These results suggest that T_SCM_ are more susceptible to infection by R5 C-HIV clinical isolates than X4 strains. Future studies involving replication-competent viruses are required to more accurately quantify the susceptibility of T_SCM_ to infection by C-HIV *in vivo*. While it is important to note that T_SCM_ represent a small proportion of the total circulating CD4^+^ T-cells and their level of infection was relatively low, demonstration of their susceptibility to infection by both R5 and X4 clinical C-HIV viruses provides new information that may help inform the design and implementation of therapies that target the T_SCM_ HIV-1 reservoir, such as anti-cancer drugs that block stem cell-specific molecular pathways expressed by T_SCM_ cells [25].

### The susceptibility of CD4^+^ T-cell subsets to CCR5-mediated infection is maintained during progression from chronic and advanced C-HIV infection

To determine whether C-HIV CD4^+^ T-cell subset tropism is altered during progression from chronic to advanced C-HIV infection, we performed intra-patient statistical analysis to compare the proportion of CD4^+^ T-cell subsets infected by Envs cloned from enrolment, intermediate and final time point plasma samples (Figure 1a). Statistical significance (by Mann Whitney test) was only observed for subject 1109 between Envs from the final time point and Envs from enrolment (E) or intermediate (I) time points, where the emergence of X4 strains was associated with a significant increase in the proportion of infected CD4^+^ T-cells that were T_N_ (E p = 0.0002, I p = 0.004), and a significant decrease in the proportion of infected T_CM_ (E p = 0.0002, I p = 0.0444), T_TM_ (E p = 0.0002, I p = 0.004), T_EM_ (E p = 0.0003, I p = 0.004) and T_SCM_ (E p = 0.0263, I p = 0.0202).

Interestingly, these results suggest that as subjects 1503 and 1854 progressed from chronic to advanced C-HIV infection over a 3 year period [12] (Table 1), circulating R5 viruses maintained their tropism profiles for CD4^+^ T-cell subsets. While additional studies involving more C-HIV Envs isolated over longer periods of time are required to confirm these findings, our data support those from a study by Buzon et al. [6], which showed that the susceptibility of T_SCM_ to infection by R5 subtype B HIV-1 is maintained during disease progression, and those from a study by Chomont et al. [1], which showed that HIV-1 can persist in T_CM_ and T_TM_ of subjects who exhibit reconstitution of CD4^+^ T-cells during suppressive antiretroviral therapy.

### Conclusions

These findings provide new insights into the natural history of tropism alterations for CD4^+^ T-cell subsets by R5 and X4 C-HIV strains during progression from chronic to advanced stages of infection. Specifically, we showed that R5 C-HIV strains maintain their preference for infecting memory CD4^+^ T-cell subsets during later stages of C-HIV infection, whereas emergent X4 C-HIV strains preferentially target T_N_. Furthermore, by demonstrating the susceptibility of T_SCM_ to infection by C-HIV, our results highlight the potential for this subset of CD4+ T-cells to serve as a long-lived viral reservoir in individuals harboring CCR5- and CXCR4-using C-HIV viruses.

## Additional file

Additional file 1:
**Extended description of Materials, Methods and Results, including the CD4** ^**+**^ **T-cell** **gating strategy, per patient CD4** ^**+**^ **T-cell** **subset distributions, CD4** ^**+**^ **T-cell** **subset CCR5 and CXCR4 expression levels, subject 1109 V3 sequence analysis, and V3 sequence accession numbers.**

## Abbreviations

(HIV-1): Human immunodeficiency virus type 1
(AIDS): Acquired immunodeficiency syndrome
(B-HIV): Subtype B HIV-1
(C-HIV): Subtype C HIV-1
(T_N_): Naïve CD4^+^ T-cell
(T_SCM_): Stem cell memory CD4^+^ T-cell
(T_CM_): Central memory CD4^+^ T-cell
(T_TM_): Transitional memory CD4^+^ T-cell
(T_EM_): Effector memory CD4^+^ T-cell
(T_EMRA_): Effector memory RA CD4^+^ T-cell
(Env): HIV-1 envelope protein
(R5): CCR5-specific HIV-1
(X4): CXCR4-specific HIV-1
(R5X4): CCR5- or CXCR4-using HIV-1
(GFP): Green fluorescent protein
(E): Plasma samples provided at enrolment
(I): 1-year later
(F): 3-years after enrolment
