# Supplementary material for: Differences in coreceptor specificity contribute to alternative tropism of HIV-1 subtype C for CD4+ T-cell subsets, including stem cell memory T-cells
Source: Retrovirology. 2014 Nov 12;11:97. doi: 10.1186/s12977-014-0097-5 (PMC4236466; doi:10.1186/s12977-014-0097-5)
Supplement: Additional file 1: — Extended description of Materials, Methods and Results, including the CD4 + T-cell gating strategy, per patient CD4 + T-cell subset distributions, CD4 + T-cell subset CCR5 and CXCR4 expression levels, subject 1109 V3 sequence analysis, and V3 sequence accession numbers. [file 12977_2014_97_MOESM1_ESM.pdf]

Supplementary Material

Differences in Coreceptor Specificity Contribute to Alternative Tropism of HIV-1 Subtype C for CD4+ T-cell Subsets, Including Stem Cell Memory T-cells.

Kieran Cashin[1,2], Geza Paukovics[3], Martin R. Jakobsen[4], Lars Østergaard[5], Melissa J. Churchill[1,6,7], Paul R. Gorry[1,2,8*] and Jacqueline K. Flynn[1,8*].

[1]Center for Biomedical Research, Burnet Institute, Melbourne, Australia 3004.
[2]Department of Microbiology and Immunology, University of Melbourne, Australia 3010.
[3]Burnet Institute Flow Cytometry Core Facility, Melbourne, Australia 3004.
[4]Department of Biomedicine, Aarhus University, Aarhus 237551, Denmark.
[5]Department of Infectious Diseases, Aarhus University, Aarhus 237551, Denmark.
[6]Department of Medicine, Monash University, Melbourne, Australia 3004.
[7]Department of Microbiology, Monash University, Melbourne, Australia 3010.
[8]Department of Infectious Diseases, Monash University, Melbourne, Australia 3004.

*Corresponding authors:
Paul R. Gorry: gorry@burnet.edu.au
Jacqueline Flynn: jflynn@burnet.edu.au

Supplementary Materials and Methods

Refer to [1] for a detailed description of the development and validation of the *in vitro* flow cytometry based infection assay used in this study.

HIV-1 Env Clones

Env clones used in this study were expressed by the pSVIII-Env mammalian expression plasmid [2]. Subtype C HIV-1 (C-HIV) Envs were previously cloned directly from plasma of subjects 1503, 1854 and 1109 and their coreceptor specificity was determined by (i) inoculating NP2 and U87 cells expressing CD4 and either CCR5 or CXCR4 with Env-pseudotyped luciferase reporter viruses [3], and (ii) the C-HIV specific genotypic algorithm $CoRSeq_{V3\text{-}C}$ [4]. The well characterised subtype B HIV-1 Envs HXB2, JR-CSF and Macs1-Spleen12 were used as controls, as previously described [5-7].

Production and Quantification of Env-pseudotyped GFP reporter viruses

Env-pseudotyped GFP reporter viruses were produced by transfecting 293T cells with pNL4-3Env-GFP and pSVIII-Env plasmids using Lipofectamine 2000 (Invitrogen, USA) at a ratio of 4:1, as previously described [1, 7]. Supernatants were harvested 48hrs later and filtered through a 0.45μm filter. Viruses were concentrated through a sucrose cushion (20% vol/vol), and stored at -80°C. The infectious units ($TCID_{50}$) of virus stocks were determined by titration in TZM-bl cells, as previously described [8, 9].

Quantifying HIV-1 infection of $CD4^+$ T-cells subsets

Peripheral blood mononuclear cells were purified from the blood of four healthy HIV-1 negative donors by density gradient centrifugation. $CD4^+$ T-cells were isolated using RosetteSep $CD4^+$ T-cell kit [(Stemcell Technologies, Canada), ensuring >95% purity of $CD3^+CD4^+$ T-cells per donor]. $CD4^+$ T-cells were resuspended in RPMI 1640 medium containing FCS (10% vol/vol) and 100µg of penicillin and streptomycin per ml, and seeded onto forty-eight well tissue culture plates (500µL at $4x10^6$ $CD4^+$ T-cells per mL). $CD4^+$ T-cells were incubated for 1hr prior to infection with 3000 infectious units of CCR5-using Env-pseudotyped GFP viruses, or 1250 infectious units of CXCR4-using viruses, by spinoculation (1200g for 2hrs) in V-bottom 96-well tissue culture plates. Reporter virus inoculums were empirically determined to be within the linear range of infection (data not shown).

Cell were then transferred to 48-well tissue culture plates and incubated for 3 days at 37^OC prior to staining with fluorochrome labelled flow cytometry antibodies (Table S2). Cells were fixed for three hours in paraformaldehyde (4% wt/vol), then washed and resuspended in FACS wash buffer (filtered PBS with 2mM EDTA and 0.5% wt/vol BSA). OneComp ebeads (eBiosciences) were used with flow cytometry antibodies as compensation controls.

HIV-1 infection was determined by $CD3^+$ and GFP^+ positivity and used to determine the distribution of infection within $CD4^+$ T-cell subsets; naïve (T_N; $CD45RO^-CCR7^+CD27^+$), stem cell memory (T_{SCM}; $CD45RO^-CCR7^+CD27^+CD95^+CD122^+$), effector memory RA ($T_{EMRA;}$ $CD45RO^-CCR7^-CD27^-$), central memory (T_{CM}; $CD45RO^+CCR7^+CD27^+$), effector memory (T_{EM}; $CD45RO^+CCR7^-CD27^-$) and transitional memory (T_{TM}; $CD45RO^+CCR7^-CD27^+$). At least 1,000,000 events were collected per donor by a LSR Fortessa flow cytometer (BD Biosciences) and analysed with Flowlogic software (Inivai Technologies). The gating strategy used here to distinguish $CD4^+$ T-cell subsets is shown in Figure S1, as described previously [1, 7].

Cells

293T cells, TZM-bl cells were maintained as previously described [7, 8].

Assembly of phenotypically characterised C-HIV V3 amino acid sequences

Previously published HIV-1 *env* third variable loop (V3) sequences were downloaded from the Los Alamos HIV Database (LANL) (http://www.hiv.lanl.gov/). V3 sequences were assigned a "X4", "CCR5-only" or "R5X4" phenotype if they were isolated from Envs documented to use CXCR4, CCR5, or either CXCR4 or CCR5 for entry into laboratory cell lines, respectively. V3 sequences were assigned a "SI (syncytia inducing)" or "NSI (non-syncytia inducing)" phenotype if they were shown to cause syncytia or not in MT2 cells, respectively. X4, R5X4 and SI V3 sequences were pooled as "CXCR4-using" sequences. CCR5-only and NSI cells were pooled as "R5" sequences. For a full list of V3 sequence accession numbers, see Appendix A.

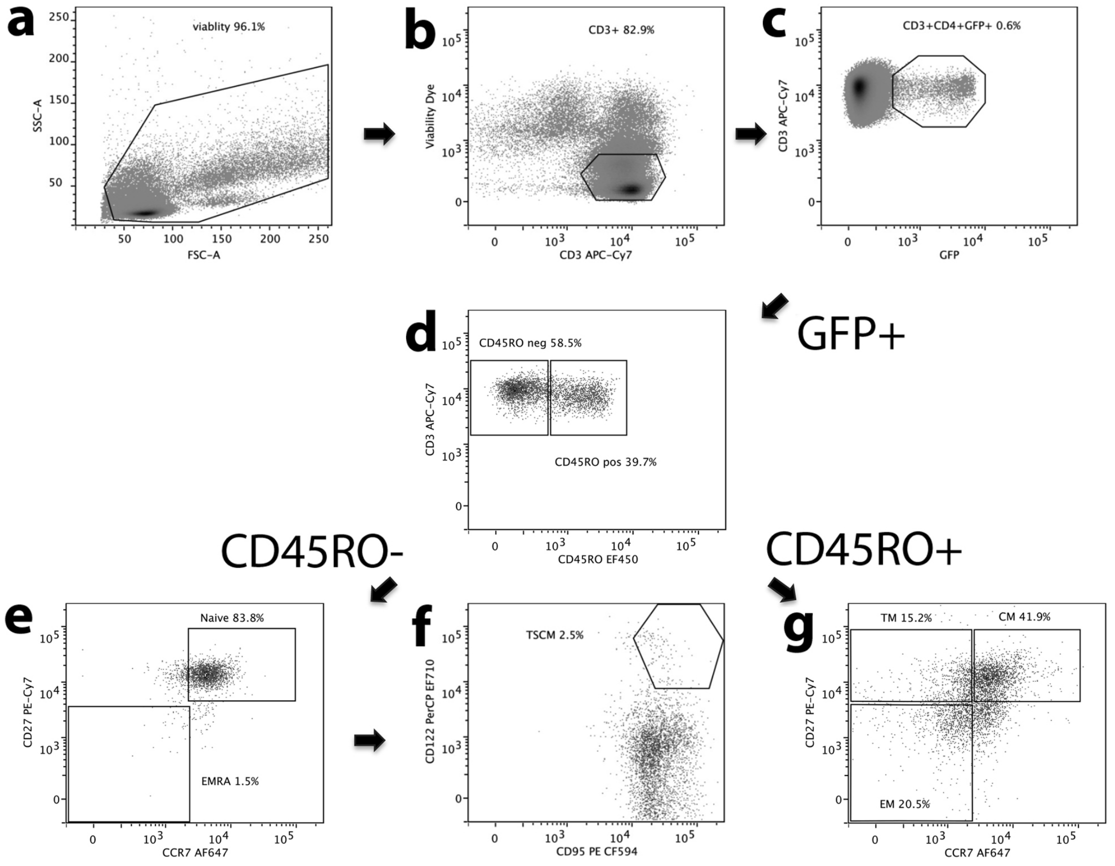

Figure S1. Gating strategy for identifying HIV-1 infected CD4$^+$ T-cell subsets. Throughout this study we used a gating strategy that was described previously by our laboratory [1]. As an example, here we have illustrated the gating strategy used to identify CD4$^+$ T-cells infected with GFP reporter virus pseudotyped with Macs1-Spleen-12 Env. CD4$^+$ T-cells were stained with a panel of flow cytometry antibodies specific for CD4$^+$ T-cell subset defining cell-surface markers. CD4$^+$ T-cells were first gated on (a) forward scatter (FSC) versus side scatter (SSC), then (b) viable CD3$^+$ T-cells (CD3 versus viability dye). HIV-1 infection was determined by CD3/GFP positivity and used to govern the distribution of infection within CD4$^+$ T cell subsets (c). (d) CD3$^+$CD4$^+$GFP$^+$ T-cells were divided into CD45RO$^+$ and CD45RO$^-$ cells. CD45RO$^-$ cells were divided into (e) naïve (T_N) and effector memory RA (T_{EMRA}) CD4$^+$ T-cells based on the expression of CCR7 and CD27. Cells in the naïve CD4$^+$ T cell gate (CD45RO$^-$CD27$^+$CCR7$^+$) were further divided into stem cell memory CD4$^+$ T cells (T_{SCM}) using CD95 and CD122 (CD45RO$^-$CCR7$^+$CD27$^+$CD95$^+$CD122$^+$), shown in plot (f). CD45RO$^+$ cells were divided into (g) central memory (T_{CM}), transitional memory (T_{TM}) and effector memory (T_{EM}) based on the expression of CCR7 and CD27. Percentages in each plot represent the proportion of the parent population that was gated, for example in (d) CD45RO$^+$ cells represent 39.7% of the viable CD3$^+$CD4$^+$ cells gated in (c) and in plot (e) naïve T cells represent 83.8% of the CD45RO$^-$ cells from plot (d). Plots (a) and (b) represent 20% of events analysed and have been reduced in these plots to show the populations more clearly.

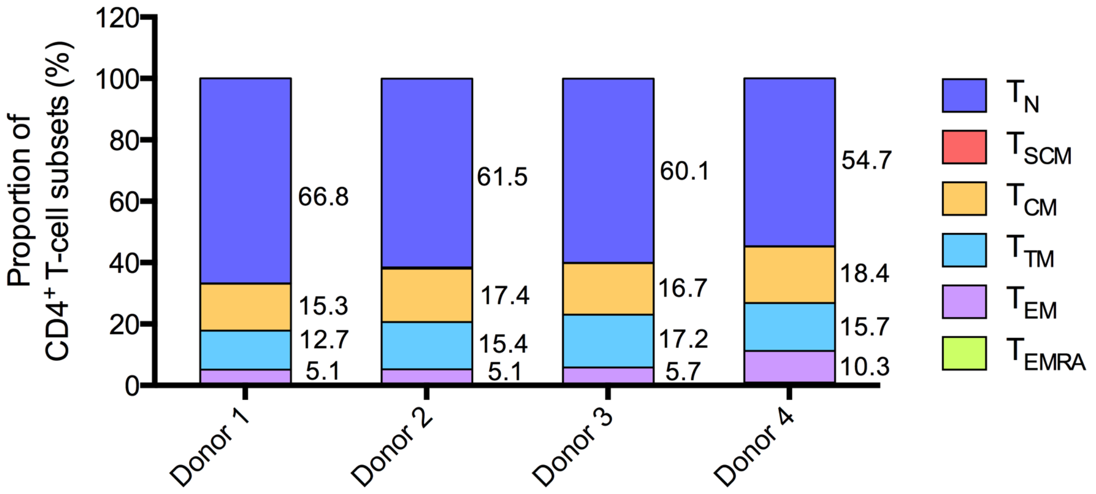

Figure S2. Proportion of CD4$^+$ T-cell subsets in each healthy donor. Values represent the percentage of CD4$^+$ T-cells that belong to the indicated subset for four healthy donors; naïve (T_N, dark blue), effector memory RA (T_{EMRA}, green), stem cell memory (T_{SCM}, red), central memory (T_{CM}, yellow), transitional memory (T_{TM}, light blue) and effector memory (T_{EM}, purple). Notably, T_{SCM} and T_{EMRA} cells were detected for each donor, yet at proportions that were <1%.

Table S1. Panel of fluorochrome labelled flow cytometry antibodies used to distinguish the $CD4^+$ T-cell subsets.

Cell-surface Marker	Fluorochrome
CD4	FITC*
CD122	EF710 (PerCPCY5.5)†
CCR7	AF-647*
CD3	APCCy7*
CD45RO	EF450 (Pacific Blue)†
Viability dye	EF506 (Amcyan)†
CD95	PE-CF594*
CD27	PE-Cy7*
CCR5	PE*
CXCR4	PE-Cy5*

†Fluorochrome labelled antibodies supplied by BD Biosciences (San Jose, USA).
*Fluorochrome labelled antibodies supplied by eBiosciences (San Diego, USA).

Table S2. Proportion of $CD4^+$ T-cells expressing CCR5 or CXCR4.

$CD4^+$ T-cell Subset	% CCR5	% CXCR4
T_N	0.5 ± 0.1	95.5 ± 2.7
T_{SCM}	4.1 ± 2.5	89.0 ± 7.4
T_{CM}	8.1 ± 3.9	87.8 ± 9.6
T_{TM}	12.6 ± 3	87.5 ±4.2
T_{EM}	19.5 ± 4	77.2 ± 11.8
T_{EMRA}	2.7 ± 2.2	91.2 ± 3.7

Values represent the percentage of $CD4^+$ T-cells within the indicated subset that express CCR5 or CXCR4, averaged across four healthy donors ± standard deviation.

Table S3. Amino acid sequences of subject 1109 Env

Subject 1109 Env	Amino Acid Sequence
**E-10**	AKTTLFCASDAKAYEKEVHNVWATHACVPTDPNPQEILLENVTENFNMWKND<br>MVDQMHDDIISIWDESLKPCVKLTPLCVTLECRKWIANTTVTWSASANQTMEG<br>EMKNCSFNVTTEIRDKIQQVSALFYKLDIVPLQENNNNYSNGSYRLINCNTSAIK<br>QACPKVAFGPIPIHYCAPAGYAILKCNNKTFNGSGPCNNVSTVQCTHGIKPVVST<br>QLLLNGSLAEEEIIIRSENLTDNAKTIIVQLKESVRIV**CTRPGNNTRKSVRIGPGQ**<br>**TFYATGEITGNIRQAHC**NISEKEWNKTLQEVGKKLREHFPNKTINFTSSSGGDL<br>EITMHSFNCRGEFFYCNTSKLFNGTYNGNDSTIKSNSTITLQCRIKQIINMWQEV<br>GRAIYAPPIAGNITCNSSITGLLLTRDGGGNDSNTETFRPGGGNMKDNWRSELY<br>KYKVVEIKPLGVAPTEAKRRVVEREKRAVGIGAVLLGFLGAAGSTMGAASITLTV<br>QARQLLSGIVQQQSNLLRAIEAQQHLLQLTVWGIKQLQTRVLAIERYLKDQQLL<br>GLWGCSGKLICTTTVPWNSSWSNRTKDYIWDNMTWMQWDREINNYTEIIYQL<br>LEESQNQQEENEKELLALDSWKNLWNWFSITNWLWYIKIFIMIVGGLIGLRIIFA<br>VLSIVNRVGQGYSPLSFQTLTPNPRGPDRLGGIEEEGGEQDRDRSVRLV
**F-30**	AKTTLFCASDAKAYEKEVHNVWATHACVPTDPNPQEILLENVTENFNMWKND<br>MVDQMHDDIISIWDESLKPCVKLTPLCVTLECRNWNASAPAWNVSADQSMEG<br>EMKNCSFNVTTEIRDKIQQVSALFYKLDIVPLQENNKNYSNGSYSYRLINCNTSVI<br>KQACPKVTFDPIPIHYCAPAGYAILKCNNKTFNGSGPCNNVSTVQCTHGIKPVVS<br>TQLLLNGSLAEEKIIIRSENLTDNAKTIIVQLKESVRIV**CTRPGNNTRKSVR*IG*IG**<br>***R*GQTFYATGDVRGDIRQAHC**NISEKEWNNTLQEVGKKLREYFPNKTINFTSSS<br>GGDLEITMHSFNCRGEFFYCNTSKLFNGTYNGTDSNSTSNATITLQCRIRQIINM<br>WQEVGRAMYAPPIAGNITCNSSITGLLLTRDGGGNNGSTETFRPGGGNMKDNW<br>RSELYKYKVVEIKPLGVAPTEAKRRVVEREKRAVGLGAVILGFLGAAGSTMGAAS<br>ITLTVQARQLLSGIVQQQSNLLRAIEAQQHLLQLTVWGIKQLQTRVLAIERYLKD<br>QQLLGLWGCSGKLICTTTVPWNSSWSNKTKDYIWDNMTWMQWDREINNYTE<br>IIYQLLEESQIQQEENEKELLALDSWKNLWNWFSITNWLWYIRIFIMIVGGLIGL<br>RIIFAVLSIVNRVRQGYSPLSFQTLTPNPRGPDRLGGIEEEGGEQDRDRSVRLV
**F-30-E10V3**	AKTTLFCASDAKAYEKEVHNVWATHACVPTDPNPQEILLENVTENFNMWKND<br>MVDQMHDDIISIWDESLKPCVKLTPLCVTLECRNWNASAPAWNVSADQSMEG<br>EMKNCSFNVTTEIRDKIQQVSALFYKLDIVPLQENNKNYSNGSYSYRLINCNTSVI<br>KQACPKVTFDPIPIHYCAPAGYAILKCNNKTFNGSGPCNNVSTVQCTHGIKPVVS<br>TQLLLNGSLAEEKIIIRSENLTDNAKTIIVQLKESVRIV**CTRPGNNTRKSVRIGPG**<br>**QTFYATGEITGNIRQAHC**NISEKEWNNTLQEVGKKLREYFPNKTINFTSSSGGD<br>LEITMHSFNCRGEFFYCNTSKLFNGTYNGTDSNSTSNATITLQCRIRQIINMWQE<br>VGRAMYAPPIAGNITCNSSITGLLLTRDGGGNNGSTETFRPGGGNMKDNWRSEL<br>YKYKVVEIKPLGVAPTEAKRRVVEREKRAVGLGAVILGFLGAAGSTMGAASITLT<br>VQARQLLSGIVQQQSNLLRAIEAQQHLLQLTVWGIKQLQTRVLAIERYLKDQQL<br>LGLWGCSGKLICTTTVPWNSSWSNKTKDYIWDNMTWMQWDREINNYTEIIYQ<br>LLEESQIQQEENEKELLALDSWKNLWNWFSITNWLWYIRIFIMIVGGLIGLRIIF<br>AVLSIVNRVRQGYSPLSFQTLTPNPRGPDRLGGIEEEGGEQDRDRSVRLV

1109-E-10 and 1109-F-30 Env V3 regions are coloured red and blue, respectively. The 1109-F-30 Ile314-Gly315 two amino acid insertion (relative to 1109-E-10) and Arg318 are coloured purple and italicized. Amino acids are number according to the HXB2 Env [10]. The 1109-F-30-E10V3 Env mutant was synthesised by GenScript Pty. Ltd (Piscataway, NJ, USA), and subcloned into the pSVIII-Env expression vector [3]. The authenticity of all Envs was verified by full-length sequencing [3].

Table S4. V3 sequence analysis.

Phenotype	Sequences (n)	Patients (n)	314-315 ins. (%)	Ile314-Gly315 (%)	GPGQ alt. (%)	GRGQ (%)	314-315 ins. & GPGQ alt. (%)	Ile314-Gly315 & GRGQ (%)
**X4**	74	20	44.6	36.5	83.8	58.1	39.2	32.4
**R5X4**	85	39	18.8	14.1	60.0	24.7	14.1	11.8
**SI**	44	6	13.6	13.6	31.8	13.6	13.6	13.6
**CXCR4-using**	**203**	**65**	**27.1**	**22.2**	**62.6**	**34.5**	**23.2**	**19.7**
**CCR5-only**	1458	271	0	0	4.7	0	0	0
**NSI**	195	85	0	0	2.6	0	0	0
**R5**	**1653**	**356**	**0**	**0**	**1.9**	**0**	**0**	**0**

V3 sequences were assigned phenotypes as described in the Supplementary Materials and Methods. X4, R5X4 and SI V3 sequences were pooled as "CXCR4-using". CCR5-only and NSI cells were pooled as "R5". Percentages represent the proportion of sequences with the indicated phenotype that exhibit the indicated V3 sequence alteration. 314-315 ins., a two amino acid insertion between at positions 314-315 of Env. Ile314-Gly315, an isoleucine and glycine insertion at positions 314-315 of Env. GPGQ alt., any V3 sequence crown motif other than GPGQ. GRGQ, a V3 sequence crown motif of GRGQ. Amino acids were numbered according to the HXB2 Env [10].

Supplementary References

1. Flynn JK, Paukovics G, Cashin K, Borm K, Ellett A, Roche M, Jakobsen MR, Churchill MJ, Gorry PR: **Quantifying susceptibility of CD4+ stem memory T-cells to infection by laboratory adapted and clinical HIV-1 strains.** *Viruses* 2014, **6:**709-726.
2. Gao F, Morrison SG, Robertson DL, Thornton CL, Craig S, Karlsson G, Sodroski J, Morgado M, Galvao-Castro B, von Briesen H, et al: **Molecular cloning and analysis of functional envelope genes from human immunodeficiency virus type 1 sequence subtypes A through G. The WHO and NIAID Networks for HIV Isolation and Characterization.** *J Virol* 1996, **70:**1651-1667.
3. Jakobsen MR, Cashin K, Roche M, Sterjovski J, Ellett A, Borm K, Flynn J, Erikstrup C, Gouillou M, Gray LR, et al: **Longitudinal Analysis of CCR5 and CXCR4 Usage in a Cohort of Antiretroviral Therapy-Naive Subjects with Progressive HIV-1 Subtype C Infection.** *PLoS One* 2013, **8:**e65950.
4. Cashin K, Gray LR, Jakobsen MR, Sterjovski J, Churchill MJ, Gorry PR: **CoRSeqV3-C: a novel HIV-1 subtype C specific V3 sequence based coreceptor usage prediction algorithm.** *Retrovirology* 2013, **10:**24.
5. Cashin K, Roche M, Sterjovski J, Ellett A, Gray LR, Cunningham AL, Ramsland PA, Churchill MJ, Gorry PR: **Alternative coreceptor requirements for efficient CCR5- and CXCR4-mediated HIV-1 entry into macrophages.** *J Virol* 2011, **85:**10699-10709.
6. De Rosa SC, Herzenberg LA, Herzenberg LA, Roederer M: **11-color, 13-parameter flow cytometry: identification of human naive T cells by phenotype, function, and T-cell receptor diversity.** *Nat Med* 2001, **7:**245-248.
7. Flynn JK, Paukovics G, Moore MS, Ellett A, Gray LR, Duncan R, Salimi H, Jubb B, Westby M, Purcell DF, et al: **The magnitude of HIV-1 resistance to the CCR5 antagonist maraviroc may impart a differential alteration in HIV-1 tropism for macrophages and T-cell subsets.** *Virology* 2013, **442:**51-58.
8. Wei X, Decker JM, Liu H, Zhang Z, Arani RB, Kilby JM, Saag MS, Wu X, Shaw GM, Kappes JC: **Emergence of resistant human immunodeficiency virus type 1 in patients receiving fusion inhibitor (T-20) monotherapy.** *Antimicrob Agents Chemother* 2002, **46:**1896-1905.
9. Yap SH, Sheen CW, Fahey J, Zanin M, Tyssen D, Lima VD, Wynhoven B, Kuiper M, Sluis-Cremer N, Harrigan PR, Tachedjian G: **N348I in the connection domain of HIV-1 reverse transcriptase confers zidovudine and nevirapine resistance.** *PLoS Med* 2007, **4:**e335.
10. Korber B FB, Kuiken C, Pillai SK, Sodroski JG: **Numbering Positions in HIV Relative to HXB2CG.** *Human Retroviruses and AIDS* 1998**:**102-111.

Appendix A: Accession Numbers

X4: AF158851, AF411966, AY230878, AY265948, AY265949, AY529666, AY529678, AY529679, DQ382362, DQ382372, DQ382378, DQ904342, DQ904343, FJ375980, FJ375983, FJ375993, FJ376006, FJ541290, FJ541293, FJ541294, FJ541295, FJ541296, FJ846628, FJ846629, FJ846630, FJ846631, FJ846632, FJ846633, FJ846634, FJ846635, FJ846636, FJ846637, FJ846640, FJ846641, FJ846643, FJ846644, FJ846645, FJ846646, FJ846647, FJ846648, FJ846649, FJ846650, FJ846651, FJ846652, FJ846653, FJ846654, FJ846655, FJ846656, FJ846657, FJ846658, FJ846659, FJ846660, KF766540, KF770411, KF770412, KF770413, KF770414, KF770415, KF770416, KF770417, KF770418, KF770419, KF770420, KF770421, KF770422, KF770423, KF770424, KF770425, KF770426, KF770427, KF770428, KF770429, KF770430, L22956.

R5X4: AF254770, AF411967, AY043174, AY230879, AY230880, AY265930, AY265931, AY265932, AY265934, AY265937, AY265942, AY265943, AY265945, AY265950, AY265951, AY529669, AY529673, AY529674, AY529677, AY887869, DQ358756, DQ382373, DQ382379, DQ904339, DQ904340, DQ904341, EF469243, EU521729, EU622014, EU760892, FJ375970, FJ375976, FJ376005, FJ376007, FJ376008, FJ376009, FJ376011, FJ376032, FJ541291, FJ541292, FJ846638, FJ846639, FJ846642, FJ846661, FJ846662, FJ977084, KF770248, KF770249, KF770250, KF770251, KF770252, KF770253, KF770254, KF770255, KF770256, KF770257, KF770258, KF770259, KF770260, KF770261, KF770262, KF770263, KF770264, KF770265, KF770266, KF770267, KF770268, KF770269, KF770270, KF770271, KF770272, KF770273, KF770274, KF770275, KF770276, KF770277, KF770278, KF770279, KF770280, KF770281, KF770282, KF770283, KF770284, KF770285, KF770286.

SI: AF056123, AF056126, AF056139, AF158871, AF158890, AF158891, AF158892, AF158893, AF158894, AF158895, AF158896, AF158897, AF158898, AF158899, AF158900, AF158901, AF158902, AF158903, AF158904, AF158905, AF158906, AF158907, AF158908, AF158909, AF158910, AF158911, AF158912, AF158913, AF158914, AF158915, AY226561, AY226562, AY226563, AY452640, AY452641, AY452642, AY452643, AY452644, AY452645, AY452646, AY452647, AY452648, AY452649, AY452650.

CCR5-only: AB023804, AF067154, AF067157, AF067158, AF153129, AF153130, AF153131, AF153132, AF153133, AF153134, AF153135, AF153136, AF153137, AF153138, AF153139, AF153140, AF153141, AF153142, AF153143, AF153144, AF153145, AF153146, AF153147, AF153148, AF153149, AF153150, AF153151, AF153152, AF153153, AF153154, AF153155, AF153156, AF153157, AF153158, AF153159, AF153160, AF153161, AF153162, AF153163, AF153164, AF153165, AF153166, AF153167, AF153168, AF153169, AF153170, AF153171, AF153172, AF153173, AF153174, AF153175, AF153176, AF153177, AF153178, AF153179, AF153180, AF153181, AF153182, AF153183, AF153184, AF153185, AF153186, AF153187, AF153188, AF153189, AF153190, AF254766, AF254767, AF254768, AF254769, AF254771, AF254772, AF254773, AF254774, AF254775, AF254776, AF254777, AF254782, AF254783, AF286227, AF286234, AF307750, AF307755, AF307756, AF321523, AF391230, AF391231, AF391232, AF391233, AF391234,

AF391235, AF391236, AF391237, AF391238, AF391239, AF391240, AF391241, AF391242, AF391243, AF391244, AF391245, AF391246, AF391247, AF391248, AF391249, AF391250, AF543910, AF543911, AF543912, AF543913, AF543920, AF544007, AF544008, AX455917, AX455929, AY043173, AY043175, AY043176, AY123262, AY123275, AY123278, AY158533, AY158534, AY158535, AY170657, AY170658, AY170659, AY170660, AY170661, AY170662, AY170663, AY170664, AY170665, AY170666, AY170667, AY228556, AY253304, AY253308, AY253322, AY255825, AY444801, AY505002, AY510057, AY510058, AY510059, AY510060, AY510061, AY510062, AY510063, AY510064, AY510065, AY510066, AY529660, AY529663, AY529676, AY585270, AY585271, AY585281, AY585282, AY585283, AY585297, AY669739, AY669741, AY669742, AY669749, AY713413, AY713414, AY713415, AY713416, AY713417, AY736822, AY736823, AY736824, AY736825, AY736826, AY833059, AY833060, AY887852, AY887853, AY887854, AY887855, AY887856, AY887857, AY887858, AY887859, AY887860, AY887861, AY887862, AY887863, AY887864, AY887865, AY887866, AY887867, AY887868, AY887870, AY887871, AY887872, AY887873, AY887874, AY887875, AY887876, AY887877, AY887878, AY887879, AY887880, AY887881, AY887882, AY887883, AY887884, AY887885, AY887886, AY887888, AY887889, AY887890, AY887891, AY887892, DQ235615, DQ235616, DQ235617, DQ235618, DQ235619, DQ235620, DQ235621, DQ235622, DQ235623, DQ235624, DQ235625, DQ235626, DQ235628, DQ235629, DQ235630, DQ235631, DQ235632, DQ235633, DQ235634, DQ235635, DQ235636, DQ235637, DQ235638, DQ235639, DQ235640, DQ235641, DQ235642, DQ235643, DQ235644, DQ235645, DQ235646, DQ235647, DQ235648, DQ358757, DQ358761, DQ358763, DQ358770, DQ382361, DQ382363, DQ382364, DQ382365, DQ382366, DQ382367, DQ382369, DQ382370, DQ382371, DQ382374, DQ382375, DQ382376, DQ382377, DQ382380, DQ388514, DQ388515, DQ388516, DQ388517, DQ411850, DQ411851, DQ411852, DQ411853, DQ411854, DQ422948, DQ435682, DQ435683, DQ435684, DQ904333, DQ904334, DQ904335, DQ904336, DQ904337, DQ904338, DQ904344, DQ904345, DQ904346, DQ904347, DQ904348, DQ904349, DQ904350, EF117265, EF117266, EF117267, EF117268, EF117269, EF117270, EF117271, EF117272, EF117273, EF117274, EU161643, EU161644, EU161645, EU293445, EU293446, EU293447, EU293448, EU293449, EU293450, EU521727, EU760888, EU760889, EU760890, EU786673, EU786681, EU855133, EU884500, EU908214, EU908215, EU908216, EU908217, EU908218, EU908219, EU908220, EU908221, EU908224, EU908225, FJ375967, FJ375968, FJ375969, FJ375971, FJ375972, FJ375973, FJ375974, FJ375975, FJ375977, FJ375978, FJ375979, FJ375981, FJ375982, FJ375984, FJ375985, FJ375986, FJ375987, FJ375988, FJ375989, FJ375991, FJ375994, FJ375995, FJ375996, FJ375997, FJ375998, FJ376001, FJ376002, FJ376003, FJ376004, FJ376012, FJ376013, FJ376014, FJ376015, FJ376016, FJ376017, FJ376018, FJ376019, FJ376020, FJ376021, FJ376022, FJ376023, FJ376024, FJ376025, FJ376026, FJ376027, FJ376028, FJ376029, FJ376030, FJ376031, FJ376033, FJ376034, FJ376035, FJ653278, FJ653279, FJ653280, FJ653281, FJ653282, FJ653283, FJ653284, FJ653285, FJ653286, FJ653287, FJ653288, FJ653289, FJ653290, FJ653291, FJ653292, FJ653293, FJ653294, FJ653295, FJ653296, FJ653297, FJ653298, FJ653299, FJ653300, FJ653301, FJ670521, FJ977078, FJ977079, FJ977080, FJ977081, FJ977082, FJ977083, FJ977085, FJ977086, FJ977087, FJ977088, FJ977089, FJ977090, FJ977091,

FJ977092, FJ977093, FJ977094, FJ977095, GU945306, GU945307, GU945308, GU945309, GU945310, GU945311, GU945312, GU945313, GU945314, GU945315, GU945316, GU945317, GU945318, GU945319, GU945320, GU945321, GU945322, GU945323, GU945324, GU945325, GU945326, GU945327, GU945328, GU945329, GU945330, GU945331, GU945332, GU945333, HM179712, HM179713, HM179714, HM179715, HM179716, HM179717, HM179718, HM179719, HM179720, HM179721, HM179722, HM179723, HM179724, HM179725, HM179726, HM179727, HM179728, HM179729, HM179730, HM179731, HM179732, HM179733, HM179734, HM179735, HM179736, HM179793, HM179794, HM179795, HM179796, HM179797, HM179798, HM179799, HM179800, HM179801, HM179802, HM179803, HM179804, HM179805, HM179806, HM179807, HM179808, HM179809, HM179810, HM179811, HM179812, HM179813, HM179814, HM179815, HM179816, HM179817, HM179818, HM179819, HM179820, HM179821, HM179822, HM179823, HM179824, HM179825, HM179826, HM179827, HM179828, HM179829, HM179830, HM179831, HM179832, HM179833, HM179834, HM179835, HM179836, HM179837, HM179838, HM179839, HM179840, HM179841, HM179842, HM179843, HM179900, HM179901, HM179902, HM179903, HM179904, HM179905, HM179906, HM179907, HM179908, HM179909, HM179910, HM179911, HM179912, HM179913, HM179914, HM179915, HM179916, HM179917, HM179918, HM179919, HM179920, HM179921, HM179922, HM179923, HM179924, HM179925, HM179926, HM179927, HM179928, HM179929, HM179930, HM179931, HM179932, HM179933, HM179934, HM179935, HM179936, HM179937, HM179938, HM179939, HM179940, HM179941, HM179942, HM179943, HM179944, HM179945, HM179946, HM179947, HM179948, HM179949, HM179950, HM179951, HM179952, HM179953, HM179954, HM179955, HM215403, HM215408, HM215411, HM215417, HM215421, HM239538, HQ678257, HQ699980, HQ699981, HQ708019, HQ708020, HQ708021, HQ708022, HQ708023, HQ708024, HQ708025, HQ708026, HQ708027, HQ708028, HQ708029, HQ708030, HQ708031, HQ708032, HQ708033, HQ708047, HQ708048, HQ708049, HQ708050, HQ708051, HQ708052, HQ708053, HQ708054, HQ708055, HQ708056, HQ708057, HQ708058, HQ708059, HQ708060, HQ708061, HQ708062, HQ708063, HQ708064, HQ708065, HQ708066, HQ708067, HQ708068, HQ708069, HQ708070, HQ708071, HQ708072, HQ708073, HQ708074, HQ708075, HQ708076, HQ708077, HQ708078, HQ708079, HQ708080, HQ708081, HQ708082, HQ708083, HQ708084, HQ708085, HQ708086, HQ708087, HQ708088, HQ708089, HQ708090, HQ708091, HQ708092, HQ708093, HQ708094, JN188292, JN687694, JN687695, JN687696, JN687697, JN687698, JN687699, JN687700, JN687701, JN687702, JN687703, JN687704, JN687705, JN687706, JN687707, JN687708, JN687709, JN687710, JN687711, JN687712, JN687713, JN687714, JN687715, JN687716, JN687717, JN687718, JN687719, JN687720, JN687721, JN687722, JN687723, JN687724, JN687725, JN687726, JN687727, JN687734, JN687737, JN687778, JN687779, JN687780, JN687781, JN687782, JN687783, JN687784, JN687785, JN687786, JN687787, JN687788, JN687789, JN687790, JN687791, JN687792, JN687793, JN687794, JN687795, JN687796, JN687797, JN687798, JN687799, JN687800, JN687801, JN687802, JN687803, JN687804, JN687805, JN687806, JN687807, JN687808, JN687809,

JN687810, JN687811, JN687812, JN687813, JN687814, JN687815, JN687816, JN687817, JN687818, JN687819, JN687820, JQ777165, JQ777166, JQ777167, JQ777168, JQ777169, JQ777170, JQ777171, JQ777172, JQ777173, JQ777174, JQ777175, JQ777176, JQ777177, JQ777178, JQ777180, JQ777181, JQ777182, JQ777183, JQ777184, JQ777185, JQ777186, JQ777187, JQ777188, JQ777190, JQ777191, JQ777192, JQ777193, JQ777194, JQ779074, JQ779075, JQ779076, JQ779077, JQ779078, JQ779079, JQ779080, JQ779081, JQ779082, JQ779083, JQ779084, JQ779085, JQ779086, JQ779087, JQ779088, JQ779089, JQ779090, JQ779091, JQ779092, JQ779093, JQ779094, JQ779095, JQ779096, JQ779097, JQ779128, JQ779129, JQ779130, JQ779131, JQ779132, JQ779133, JQ779134, JQ779135, JQ779136, JQ779137, JQ779138, JQ779139, JQ779140, JQ779141, JQ779142, JQ779143, JQ779144, JQ779145, JQ779146, JQ779147, JQ779170, JQ779171, JQ779172, JQ779173, JQ779174, JQ779175, JQ779176, JQ779177, JQ779178, JQ779179, JQ779180, JQ779181, JQ779182, JQ779183, JQ779184, JQ779185, JQ779186, JQ779187, JQ779188, JQ779189, JQ779190, JQ779191, JQ779192, JQ779193, JQ779194, JQ779195, JQ779196, JQ779197, JQ779198, JQ779199, JQ779200, JQ779201, JQ779202, JQ779203, JQ779204, JQ779205, JQ779226, JQ779227, JQ779228, JQ779229, JQ779230, JQ779231, JQ779232, JQ779233, JQ779234, JQ779235, JQ779236, JQ779237, JQ779238, JQ779239, JQ779240, JQ779241, JQ779242, JQ779243, JQ779244, JQ779245, JQ779246, JQ779247, JQ779248, JQ779249, JQ779250, JQ779251, JQ779252, JQ779253, JQ779254, JQ779255, JQ779286, JQ779287, JQ779288, JQ779289, JQ779290, JQ779291, JQ779292, JQ779293, JQ779294, JQ779295, JQ779296, JQ779297, JQ779298, JQ779299, JQ779300, JQ779301, JQ779302, JQ779303, JQ779304, JQ779305, JQ779306, JQ779307, JQ779308, JQ779309, JQ779310, JQ779311, JQ779312, JQ779313, JQ779314, JQ779315, JQ779316, JQ779317, JQ779318, JQ779319, JQ779320, JQ779321, JQ779322, JQ779323, JQ779324, JQ779884, JQ779885, JQ779886, JQ779887, JQ779888, JQ779889, JQ779890, JQ779891, JQ779893, JQ779895, JQ779896, JQ779897, JQ779898, JQ779899, JQ779900, JQ779901, JQ779902, JQ779903, JQ779904, JQ779905, JQ779906, JQ779907, JQ779908, JQ779909, JQ779910, JQ779911, JQ779912, JQ779913, JX140662, JX140663, JX140664, JX140665, JX140666, JX140667, JX140668, JX140669, JX508863, JX508864, JX508865, JX508873, JX508874, JX972455, JX972456, JX972457, JX972458, JX972459, JX972460, JX972461, JX972462, JX972463, JX972464, JX972465, JX972466, JX972467, JX972468, JX972469, JX972470, JX972471, JX972472, JX972473, JX972474, JX972475, JX972476, JX972477, JX972478, JX972479, JX972480, JX972481, JX972482, JX972483, JX972484, JX972485, JX972486, JX972487, JX972838, JX972839, JX972840, JX972841, JX972842, JX972843, JX972844, JX972845, JX972931, JX972932, JX972933, JX972934, JX972935, JX972936, JX972937, JX972938, JX972939, JX972940, JX972941, JX972942, JX972943, JX972944, JX972945, JX972946, JX972947, JX972948, JX972949, JX972950, JX972951, JX972952, JX972953, JX972954, JX972955, JX972956, JX972957, JX972958, JX972959, JX972960, JX972961, JX972962, JX972963, JX972964, JX972986, JX972987, JX972988, JX972989, JX972990, JX972991, JX972992, JX972993, JX972994, JX972995, JX972996, JX972997, JX972998, JX973075, JX973076, JX973077, JX973078, JX973079, JX973080, JX973081, JX973082, JX973083, JX973084, JX973085, JX973086, JX973087, JX973088, JX973089, JX973090, JX973091, JX973092, JX973093, JX973094, JX973095, JX973096, JX973097, JX973098, JX973099, JX973100,

JX973101, JX973171, JX973172, JX973173, JX973174, JX973175, JX973176, JX973177, JX973178, JX973179, JX973180, JX973234, JX973235, JX973236, JX973237, JX973238, JX973239, JX973240, JX973241, JX973242, JX973243, JX973244, JX973245, JX973246, JX973247, JX973248, JX973249, JX973250, JX973251, JX973252, JX973253, JX973254, JX973255, JX973256, JX973257, JX973258, JX973259, JX973260, JX973261, JX973262, JX973263, JX973264, JX973265, JX973266, JX973267, JX973268, JX973269, JX973270, KC156114, KC156115, KC156116, KC156117, KC156118, KC156119, KC156120, KC156121, KC156122, KC156123, KC156124, KC156125, KC156126, KC156127, KC156128, KC156129, KC156130, KC156212, KC156213, KC156214, KC156215, KC156216, KC156217, KC156218, KC156219, KC156220, KC156221, KC156222, KC156223, KC156224, KC156225, KC156226, KC156227, KC156228, KC156229, KC156230, KC156231, KC156232, KC156233, KC156234, KC156235, KC156237, KC156238, KC156239, KC156240, KC156241, KC156242, KC156243, KC156244, KC156245, KC156247, KC156248, KC156249, KC156250, KC156251, KC156269, KC156270, KC156271, KC156272, KC156273, KC156274, KC156275, KC156277, KC156278, KC156279, KC156280, KC156281, KC156282, KC156283, KC156284, KC156285, KC156286, KC156287, KC156288, KC156289, KC156290, KC156291, KC156293, KC156294, KC156295, KC156296, KC156318, KC156319, KC156320, KC156321, KC156322, KC156323, KC156324, KC156325, KC156326, KC156327, KC156328, KC156329, KC156330, KC156331, KC156332, KC156333, KC156334, KC156335, KC156336, KC156337, KC156338, KC156339, KC156340, KC156341, KC156342, KC156343, KC156372, KC156373, KC156374, KC156375, KC156376, KC156377, KC156378, KC156379, KC156381, KC156383, KC156384, KC156385, KC156386, KC156387, KC156388, KC156389, KC156390, KC156391, KC156392, KC156393, KC156394, KC156395, KC156396, KC156397, KC156398, KC156399, KC156400, KC156401, KC156431, KC156432, KC156433, KC156434, KC156435, KC156436, KC156437, KC156438, KC156439, KC156440, KC156441, KC156442, KC156443, KC156444, KC156445, KC156446, KC156447, KC156448, KC156449, KC156450, KC156451, KC156452, KC156453, KC156454, KC156455, KC156456, KC156457, KC156458, KC156460, KC156461, KC156462, KC156464, KC156465, KC156466, KC156467, KC156468, KC156469, KC156470, KC156471, KC473844, KF716466, KF716467, KF766537, KF766538, KF766539, KF766541, KF770287, KF770288, KF770289, KF770290, KF770291, KF770292, KF770293, KF770294, KF770295, KF770296, KF770297, KF770298, KF770299, KF770300, KF770301, KF770302, KF770303, KF770304, KF770305, KF770306, KF770307, KF770308, KF770309, KF770310, KF770311, KF770312, KF770313, KF770314, KF770315, KF770316, KF770317, KF770318, KF770319, KF770320, KF770321, KF770322, KF770323, KF770324, KF770325, KF770326, KF770327, KF770328, KF770329, KF770330, KF770331, KF770332, KF770333, KF770334, KF770335, KF770336, KF770337, KF770338, KF770339, KF770340, KF770341, KF770342, KF770343, KF770344, KF770345, KF770346, KF770347, KF770348, KF770349, KF770350, KF770351, KF770352, KF770353, KF770354, KF770355, KF770356, KF770357, KF770358, KF770359, KF770360, KF770361, KF770362, KF770363, KF770364, KF770365, KF770366, KF770367, KF770368, KF770369, KF770370, KF770371, KF770372, KF770373, KF770374, KF770375, KF770376, KF770377, KF770378, KF770379, KF770380, KF770381, KF770382, KF770383, KF770384, KF770385, KF770386, KF770387, KF770388, KF770389, KF770390, KF770391, KF770392, KF770393, KF770394, KF770396, KF770397, KF770398, KF770399, KF770400, KF770401,

KF770402, KF770403, KF770404, KF770405, KF770406, KF770407, KF770408, KF770409, KF770410, KF770431, KF770432, KF770433, KF770434, KF770435, KF770436, KF770437, KF770438, KF770439, KF770440, KF770441, KF770442, KF770443, KF770444, KF770445, KF770446, KF770447, KF770448, KF770449, KF770450, KF770451, KF770452, KF770453, KF770454, KF770455, L22940, L48068, U08453, U08454, U08455, U08720, U08785, U09126, U09132, U09133, U15121, U45485, U52953.

NSI: AF158843, AF158844, AF158845, AF158846, AF158847, AF158848, AF158849, AF158850, AF158852, AF158853, AF158854, AF158855, AF158856, AF158857, AF158858, AF158859, AF158860, AF158861, AF158862, AF158863, AF158864, AF158865, AF158866, AF158867, AF158868, AF158869, AF158870, AF158872, AF158873, AF158874, AF158875, AF158876, AF158877, AF158878, AF158879, AF158880, AF158881, AF158882, AF158883, AF158884, AF158885, AF158886, AF158887, AF158888, AF158889, AY135934, AY135935, AY135936, AY135937, AY135938, AY135939, AY135940, AY135941, AY135942, AY135943, AY135944, AY135945, AY135946, AY135947, AY135948, AY135970, AY135971, AY135972, AY135973, AY135974, AY135975, AY135976, AY135977, AY135978, AY135979, AY135980, AY135981, AY135982, AY135983, AY135984, AY135986, AY135987, AY135988, AY135989, AY135990, AY135991, AY135992, AY135993, AY135994, AY135995, AY135996, AY135997, AY135998, AY135999, AY136000, AY136001, AY136002, AY136003, AY136004, AY136005, AY136006, AY136007, AY136008, AY136009, AY136010, AY136011, AY136012, AY136013, AY136014, AY136015, AY136016, AY136017, AY136018, AY136019, AY136020, AY136021, AY136022, AY136023, AY136024, AY136025, AY136026, AY136027, AY136028, AY136029, AY136030, AY136031, AY136032, AY136033, AY136034, AY136035, AY136036, AY136037, AY136038, AY136039, AY136040, AY136041, AY136042, AY136043, AY136044, AY136045, AY136046, AY136047, AY136048, AY136049, AY136050, AY136051, AY136052, AY136053, AY136054, AY136055, AY136056, AY136057, AY136058, AY136059, AY136060, AY136061, AY136062, AY136063, AY136064, AY136065, AY136066, AY136067, AY136068, AY136069, AY136070, AY136071, AY265929, AY265933, AY265935, AY265936, AY265938, AY265939, AY265940, AY265941, AY265944, AY265946, AY265947, AY265952, AY265953, AY265954, AY265955, AY265956, AY265957, AY265958, AY529667, GQ926904, GQ926909, L48067, U58785, U58786, U58788, U58789, U58790, U58791, U58793, U58794, U58795, U58796, U58797, U58798.
